# The effect of removing hearing aids on postural sway in older adults with age-related hearing loss: an experimental study

**DOI:** 10.3389/fnagi.2025.1534227

**Published:** 2025-03-24

**Authors:** Sylwia Kolasa, Bård Bogen, Roy M. Nilsen, Frederik Goplen, Stein Helge G. Nordahl, Kjersti Thulin Wilhelmsen, Jan Erik Berge, Dara Meldrum, Susanne S. Hernes, Ole Martin Steihaug, Liv H. Magnussen

**Affiliations:** ^1^Department of Health and Function, Western Norway University of Applied Science, Bergen, Norway; ^2^Department for Rehabilitation Services, Haraldsplass Deaconess Hospital, Bergen, Norway; ^3^Department of Otorhinolaryngology and Head and Neck Surgery, Haukeland University Hospital, Bergen, Norway; ^4^Department of Clinical Medicine, University of Bergen, Bergen, Norway; ^5^Academic Unit of Neurology, Trinity College Dublin, Dublin, Ireland; ^6^Department of Internal and Geriatric Medicine, Sorlandet Hospital Arendal, Arendal, Norway

**Keywords:** older adults, hearing loss, hearing aids, balance, postural control, posturography, postural sway

## Abstract

**Background:**

Studies show that there is an association between age-related hearing loss (HL) and balance in older individuals. Several studies have indicated that the use of hearing aids (HAs) may have a positive impact on balance. However, the effect of HAs on postural sway in standing is still debated and unclear. The aim of this study was to examine differences in postural sway with and without the use of HAs, and the association between hearing threshold on balance and controlling for confounders, when comparing the use of HAs to not using them.

**Methods:**

In this study, balance was tested in standing position on a force platform in individuals ≥70 years (*N* = 50) with HL (>30 dB) under four conditions (on a firm surface with eyes open and closed, and on a foam surface eyes open and closed). Postural sway was registered with and without using HAs, and the difference between the two conditions was examined by paired sample t-test. Associations between postural sway and hearing threshold was examined separately with and without using HAs by multiple regression analysis.

**Results:**

There was a statistically significant reduced postural sway (better balance) on a firm surface with eyes open with an effect size of 0.43 (95% CI 0.15 to 0.73, *p* = 0.003) using HAs compared to not using them. Multiple regression analyses did not show any significant associations between postural sway and hearing threshold after adjustments for cofounding factors, including age, sex, education, diabetes, cardiovascular diseases, and dizziness.

**Discussion:**

In this study, participants demonstrated significantly better balance when standing on a firm surface with eyes open while using HAs, compared to standing without them. However, this improvement was not observed when standing on foam surface. Further research is necessary to examine the impact of HAs on balance across various conditions and surfaces. Future studies should also investigate the underlying mechanisms of these effects, including how HAs may influence proprioception and postural control, particularly in environments that challenge balance, such as foam surfaces.

## 1 Introduction

Presbycusis is gradual age-related hearing loss (HL) and caused by the natural degeneration of the auditory system over time. Usually, age-related HL affects both ears equally because the changes occur in both ears at the same rate (Peate and Wild, 2018).

HL is the third most prevalent chronic health disease among older adults and is ranked as the second leading cause of years lived with disability (Haile et al., 2021). It has been estimated that 71% of those over 70 years have some level of hearing impairment (Slade et al., 2020). HL has been found to be associated with several comorbidities such as physical health decline, anxiety, depression, isolation, cognitive decline, and dementia (Haile et al., 2021; Engdahl et al., 2021; Sun et al., 2021). Additionally, HL has been found to be associated with imbalance and increased risk of falls (Viljanen et al., 2009). Carpenter and Campos (2020) proposed that theories explaining this association include: (a) age-related decline in labyrinth function, where HL serves as an indicator of vestibular hypofunction contributing to imbalance, and (b) the maintenance of postural stability relies on sensory inputs from visual, auditory, vestibular, and somatosensory sources. Thus, the absence of auditory cues in individuals with age-related HL directly affects balance (Viljanen et al., 2009; Rumalla et al., 2015). Since falls are considered the most common cause of injuries in older populations, many leading to severe consequences such as hospitalization (Sharif et al., 2018), it is important to understand the contribution of HL to postural instability. Postural sway, the natural side-to-side movement of the body while standing, is linked to an increased risk of falls in older people (Johansson et al., 2019) Excessive or unstable postural sway can indicate poor balance, making individuals more prone to losing balance and falling (Sharif et al., 2018). Therefore, postural sway is an important predictor of fall risk, particularly when combined with factors like muscle weakness, sensory impairments, or medication (Sharif et al., 2018). Normally, maintaining balance involves visual, vestibular, and somatosensory inputs. Lubetzky et al. (2020) suggest that individuals rely on different sensory inputs based on availability of these inputs and task demands. Studies indicate that auditory input also affects postural sway but its impact is minor compared to other sensory systems. Auditory cues become more important when there is sensory loss, such as in cases of vestibular dysfunction or visual impairment, but are less crucial in healthy adults who can use multiple strategies for maintaining balance.

Since mild and moderate HL is often not treatable through surgical or medical interventions, provision of hearing aids (HAs) is a standard treatment for older individuals with HL (Cox et al., 2014). HAs amplify and transmit sound to the inner ear. Several studies have shown that using HAs can improve balance, cognitive function, quality of life, and reduce listening efforts (Ernst et al., 2021; Dawes et al., 2015; Atef et al., 2023; Sanders et al., 2021; Yang et al., 2022; Bainbridge and Wallhagen, 2014). However, systematic reviews by Mahafza et al. (2022) and Borsetto et al. (2021) conclude that there is a lack of high-quality studies investigating the effect of HAs on standing balance, and amplification of sound through HAs have not always been found to improve standing balance. They also suggested that future studies should consider confounders such as age, and degree of HL.

The aim of this study was to assess standing balance measured by postural sway in older adults with age-related HL (>30 dB), with and without the use of HAs. Additionally, we also aimed to examine the association between hearing threshold in better ear (pure tone audiometry, PTA) on standing balance while controlling for age, sex, diabetes, cardiovascular disease, and self-reported dizziness, factors that may influence this association. We hypothesized that standing balance in older adults would be better when using HAs compared to not using them.

## 2 Materials and methods

This experimental study was conducted at the SimArena movement laboratory at the Western Norway University of Applied Sciences (HVL) in Bergen, between September 2020 and June 2021. People eligible for study participation had to be over 70 years of age and have age-related HL (>30 dB) confirmed by general practitioners or audiologists. They also had to be users of HAs. Exclusion criteria included HL not attributed to the aging process, previous ear surgery, neurological conditions like Parkinson’s or stroke, and medical conditions that could significantly influence gait and balance, such as head or ear injuries. Various sources were utilized for participant recruitment, including the Hearing Center at Haukeland University Hospital in Bergen, general practitioners from the Norwegian Primary Care Research Network who referred eligible patients (Kristoffersen et al., 2022), the Norwegian Association of the of the Hearing Impaired (HLF), which shared information through its newsletter, and retired staff members at HVL.

### 2.1 Ethics

The study adhered to the criteria and principles in the Declaration of Helsinki and was approved by the Regional Committee for Medical Research Ethics of South-East Norway (REK Sør-Øst D 33195) as well as the Norwegian Centre of Research Data (NSD 167090). All participants gave their written, informed consent. The study was carried out in accordance with the STROBE checklist for cross-sectional studies (Vandenbroucke et al., 2007) and was registered on Clinical Trials.gov (NCT04283279).

### 2.2 Procedures and data collection

The assessment of standing balance was conducted at the SimArena movement laboratory within a controlled environment characterized by the absence of ambient sounds or conversations. All tests were administered by the first author (SK), who is an experienced audiologist and a qualified physiotherapist.

### 2.3 Measurements

#### 2.3.1 Demographic and epidemiological data collection

To collect demographic and epidemiological data, a self-constructed questionnaire was used. The questionnaire included questions about age, sex, education, and the presence of specific medical conditions such as diabetes, cardiovascular diseases, nervous system diseases, ear-related ailments, and self-reported dizziness. Single dichotomous questions (yes/no) were asked to determine whether the respondents had diabetes or cardiovascular diseases, or if they had experienced dizziness.

### 2.4 Main outcome

#### 2.4.1 Standing balance measured by posturography (postural sway)

Postural sway during quiet standing was assessed by a commercially available force platform (BTG4, HUR health). The BTG4 Force Platform provides high-frequency sampling, ensuring precise data capture for dynamic movements and balance assessments. It is widely used in clinical settings and research studies focused on balance, postural control, and rehabilitation for individuals with neurological conditions and sports injuries (Blosch et al., 2019). With high-precision sensors measuring forces in three directions, it offers reliable assessments of ground reaction forces and center of pressure. Although direct comparisons to other platforms are limited, its accuracy is typically within 1–2%. Additionally, it is portable with integrated software, making it effective for both clinical and field studies (Blosch et al., 2019). Sway was registered as trace length (in millimeters) of the center of pressure (COP) recorded when standing quietly for 30 s. Participants were instructed to stand with their arms crossed and heels positioned 2 cm apart, under four conditions; firm surface with eyes open (EO firm) and closed (EC firm), and soft surface (6 cm thick Airex foam pad) with eyes open (EO foam) and closed (EC foam) (Blosch et al., 2019). The order of the testing conditions was fixed, and all participants were tested first while wearing their HAs and subsequently without HAs. Previous studies have demonstrated acceptable validity and reliability of posturography in similar settings (Blosch et al., 2019).

### 2.5 Exposure

#### 2.5.1 Hearing threshold

The hearing threshold was assessed using a portable screening audiometer for manual pure tone audiometry (MADSEN Micromate 304 with TDH-39 supra-aural audiometric headphones). Air-conduction pure-tone audiometry (PTA) was conducted at four frequencies: 0.5, 1, 2, and 3 kHz, measured in dB hearing level (HL) in line with recommendations (Gurgel et al., 2012). The PTA values were calculated separately calculated for each ear, as recommended by the Hearing Committee of the American Academy of Otolaryngology – Head and Neck Surgery (Gurgel et al., 2012). The better ear was selected for analysis, as it has been demonstrated to have a stronger association with physical performance (Berge et al., 2019; Kolasa et al., 2024). Hearing assessments were conducted in a quiet environment. Sensitivity and specificity of audiometry has been found to be acceptable (Ting and Huang, 2023).

### 2.6 Co-variates

To examine the differences in postural sway with and without HAs, a comprehensive set of covariates was collected, which included age, sex, education, diabetes, cardiovascular disease, self-reported dizziness, cognitive function. The selection of covariates was done by making directed acyclic graphs (DAGs), which can minimize bias in research. We used the software DAGitty^1^ to get a clearer view of how the different variables may be associated with one another and made the regression models based on this (Textor et al., 2011).

### 2.7 Further measurements

#### 2.7.1 The dizziness handicap inventory

The DHI is a questionnaire designed to evaluate the impact of dizziness on a person’s daily life (Jacobsen, 1990). The DHI contains 25 questions, with ratings “0” for no, “2” for sometimes, and “4” for yes, giving scores between 0 and 100, with higher scores indicating higher disability. The questionnaire has been translated and cross-culturally validated in a Norwegian population (DHI-N), demonstrating satisfactory measurement properties (Tamber et al., 2009). A cut-off for disability is suggested to be 29 DHI points (Tamber et al., 2009). The DHI-N was included as background information and to assesses the self-perceived impact of dizziness on an individual’s daily life.

#### 2.7.2 The World Health Organization disability assessment schedule 2.0, 12-item version (WHODAS 2.0 12)

The World Health Organization Disability Assessment Schedule 2.0, 12-item version (WHODAS 2.0 12) is a questionnaire designed to evaluate difficulties due to health conditions (Saltychev et al., 2021), its total score range is from “12” to “60.” Health conditions include diseases or illnesses, other health problems that may be short or long lasting, injuries, mental or emotional problems, and problems with alcohol or drugs. WHODAS 2.0 12 contains 12 questions, each item ranged from “1” to “5” to indicate the level of difficulty or a problem (Saltychev et al., 2021). The scoring is scaled in a negative direction, which means that a higher score indicates a lower quality of life. The overall points for global disability therefore ranged from 12 (no disability) to 60 (complete disability), with higher results indicating a higher level of disability (Saltychev et al., 2021). The WHODAS 2.0 was included as background information to illustrate the physical functioning of the participants.

#### 2.7.3 The trail making test (TMT)

The Trail Making Tests (TMT) A and B evaluate cognitive domains such as executive function, visual scanning, and working memory by requiring participants to connect a series of 25 circles in a specified order. TMT A assesses processing speed through a straightforward numerical sequencing task, while TMT B challenges participants to alternate between numbers and letters, thereby engaging executive functioning (Rasmusson et al., 1998). Completion times for TMT A typically range from 40 to 60 s for cognitively healthy older adults, whereas TMT B average between 60 and 90 s (Ashendorf et al., 2008).

### 2.8 Analysis

The statistical analyses were conducted using IBM SPSS Statistics 27. Means and standard deviations were used for normally distributed variables, while medians and interquartile range (IQR) where used for non-normally distributed variables. The dependent variables were postural sway measures (EOfrim, ECfirm, EOfoam, ECfoam). Since these variables were not normally distributed, they were logarithmically transformed to improve normality. Paired-samples t-tests were used to examine the differences in postural sway with and without HAs. Multiple linear regression analysis was used to assess the association between PTA better ear and postural sway. The independent variable in the regression analyses was hearing threshold (PTA, better ear). All regression analyses were performed in both unadjusted models and models adjusted for various confounding factors. Model 1 included age, sex, and education, Model 2 included Model 1 along with diabetes, cardiovascular diseases, and self-reported dizziness. The regression analyses were performed with and without using HAs to evaluate whether there was difference in the associations in such conditions. The PTA better ear was divided by 10, and the regression coefficients, therefore, should be understood as change in balance per 10-unit increase in PTA.

## 3 Results

Fifty participants with HL were examined. Mean age was 76.2 years (SD = 4.8), 60% were female, and the educational level was overall high (Table 1). The mean PTA in the best ear was 47.3 dB (SD = 11.3). The median scores of TMT A and B were 46 and 100 s respectively, within the age adjusted limits of 48 and 100 s, indicating that there were no signs of cognitive decline in our sample. The median score of WHODAS was 13.5 (IQR = 12.0–17.0), suggesting a minimal impact on the overall physical functioning and disability. The median DHI-N scores was 8, thus far below the 29-point cut-off value for severe disability (Table 1).

**Table 1:** Distribution of demographics and comorbidities in persons with HL (*N* = 50).

Characteristic	Values
Age (years), mean (SD), min-max	76.2 (4.8), 70–90
Sex, *N* (%)	
Men	20 (40)
Women	30 (60)
Education, *N* (%)	
High school	9 (18)
University	41 (82)
Diabetes, *N* (%)	
No	45 (90)
Yes	5 (10)
Cardiovascular diseases, *N* (%)	
No	48 (96)
Yes	2 (4)
Dizziness, *N* (%)	
No	31 (62)
Yes	19 (38)
PTA, mean (SD), min-max	51.1 (11.2), 20.6–86.3
Better ear, min-max	47.3 (11.3), 18.8–83.3
Worse ear, min-max	55 (11.9), 22.5–88.8
TMT-A (40–60 s.), median (IQR), min-max	45 (32–58.5), 23–98
TMT-B (60–90 s.), median (IQR), min-max	100.5 (79.8–152.3), 49–300
DHI-N total (0–100), median (IQR), min-max	0.0 (0.0–8.0), 0.0–74
WHODAS 2.0 12 (0–60), median (IQR), min-max	13.5 (12.0–17.0), 11–37

PTA, Pure Tone Audiometry. TMT-A, rail Making Test A. TMT-B, Trail Making Test B. DHI-N, Dizziness Handicap Inventory – Norwegian version. WHODAS 0.2 12, World Health Organization Disability Assessment Schedule 2.0, 12-item version. IQR = 25th-75th percentile.

**Figure 1:**
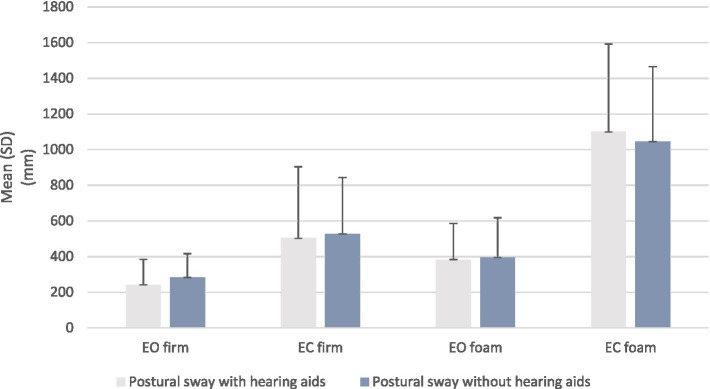
Distribution of postural sway on a firm and foam surface with eyes open and closed in older persons with HL (*N* = 50). Bars present SD. Postural sway is measured in millimeters (mm).

Figure 1 is a graphical presentation of the test results for postural sway with and without the use of HAs and illustrates visible difference in balance when participants were standing on a firm surface with eyes open while using HAs. Please see Supplementary Table 1s.

The paired-samples t-test revealed that there was a statistically significant increase in postural sway on firm surface with eyes open (*p* = 0.003) when the HAs were not used. There were no other significant differences in the other test conditions (Table 2).

**Table 2:** Difference in log-transformed postural sway with and without HAs, analyzed by paired-samples *t*-test (*N* = 50).

Postural sway in millimeters (mm) Log-transformed	With hearing aids mean (SD)	Without hearing aids mean (SD)	Mean difference (95% CI) *p*-value^a^	Effect size (Cohen’s d)
Eyes open				
Firm	5.38 (0.45)	5.54 (0.46)	0.16 (0.058 to 0.265) *p* = 0.003	0.43 (0.150 to 0.731)
Foam	5.87 (0.37)	5.87 (0.45)	0.00 (−0.096 to 0.099) *p* = 0.969	0.01 (−0.272 to 0.283)
Eyes closed				
Firm	6.01 (0.60)	6.13 (0.52)	0.11 (−0.010 to 0.240) *p* = 0.071	0.26 (−0.022 to 0.541)
Foam	6.91 (0.45)	6.88 (0.38)	−0.03 (−0.120 to 0.062) *p* = 0.528	−0.09 (−0.367 to 0.188)

a P-value for difference (paired-samples *t*-test).

Multiple linear regression analysis did not show significant associations between hearing threshold (PTA better ear) and postural sway after adjustment for age, sex, education, diabetes, cardiovascular diseases and dizziness (Table 3).

**Table 3:** Association between hearing threshold (PTA better ear) and postural sway with and without HAs (*N* = 50).

Postural sway in millimeters (mm) Log-transformed	Crude model	Model 1	Model 2
	Coefficient (95% CI) *p*-value	Coefficient (95% CI) *p*-value	Coefficient (95% CI) *p*-value
With hearing aids			
Eyes open			
Firm	0.059 (−0.055 to 0.174) *p* = 0.300	0.034 (−0.075 to 0.143) *p* = 0.535	0.028 (−0.082 to 0.137) *p* = 0.612
Foam	0.030 (−0.066 to 0.126) *p* = 0.533	0.005 (−0.087 to 0.097) *p* = 0.912	−0.002 (−0.095 to 0.092) *p* = 0.970
Eyes closed			
Firm	0.123 (−0.026 to 0.273) *p* = 0.104	0.077 (−0.068 to 0.222) *p* = 0.288	0.062 (−0.079 to 0.204) *p* = 0.381
Foam	−0.007 (−0.123 to −0.108) *p* = 0.897	−0.026 (−0.149 to 0.097) *p* = 0.672	−0.033 (−0.145 to −0.078) *p* = 0.551
Without hearing aids			
Eyes open			
Firm	0.010 (−0.108 to 0.128) *p* = 0.862	−0.016 (−0.130 to 0.097) *p* = 0.774	−0.028 (−0.142 to 0.086) *p* = 0.625
Foam	0.047 (−0.067 to 0.162) *p* = 0.411	0.029 (−0.088 to 0.146) *p* = 0.622	0.014 (−0.104 to 0.133) *p* = 0.809
Eyes closed			
Firm	0.014 (−0.119 to 0.147) *p* = 0.835	−0.046 (−0.166 to 0.075) *p* = 0.448	−0.063 (−0.178 to 0.052) *p* = 0.273
Foam	0.044 (−0.054 to 0.142) *p* = 0.370	0.017 (−0.084 to 0.118) *p* = 0.732	0.015 (−0.087 to 0.117) *p* = 0.770

Model 1: adjusted for age, sex, education; Model 2: adjusted for age, sex, education, diabetes and cardiovascular disease, and dizziness. *p* < 0.05.

## 4 Discussion

In this study, we investigated the effect of HAs use on standing balance, registered by postural sway, in 50 older individuals with age-related HL. A significant reduction in postural sway (better balance) was found when participants used HAs while standing on a firm surface with eyes open compared with not using them. This contrasts with the results reported by Ninomiya et al. (2021), Negahban and Nassadj (2017), and Rumalla et al. (2015), which demonstrated significant improvements in standing balance on foam surfaces when participants used HAs, regardless of whether their eyes were open or closed. No significant differences were observed for other test conditions (firm EO and foam EO/EC), and no significant correlation was found between PTA in better ear and postural sway across any conditions, regardless of HAs use.

The main finding in this study was that postural sway improved in the least challenging test condition—standing on a firm surface with eyes open—while using HAs, without any auditory cues. This condition allows the use of multiple sensory inputs, including vision, vestibular, and proprioception. This suggests that HAs may play a role in balance maintenance, but only in less demanding conditions without auditory cues. Conversely, when the other senses were challenged in conditions such as eyes closed on a firm surface (no vison), eyes open on a foam surface (disturbed proprioception), and eyes closed on a foam surface (no vison and disturbed proprioception), hearing appeared to have a less dominant role. These results provide insights into the complex interplay between sensory feedback mechanisms and postural sway, highlighting the limitations of the human postural control system in response to environmental challenges (Peterka, 2002). This may explain the difficulties the participants demonstrated in our study when the testing conditions became more challenging.

Negahban and Nassadj (2017) highlighted that, in addition to visual, vestibular, and somatosensory inputs, auditory inputs should be considered important contributors in maintaining postural stability. Rumalla et al. (2015) also found reduced sway variability in the presence of auditory white noise, which masked background sounds that could potentially disrupt participants’ focus during balance tasks. This is consistent with our findings, suggesting that using HAs plays a role in improving postural stability under optimal conditions even without white noise, specifically when participants are instructed to maintain their balance with their eyes open on a firm surface. This scenario appears to provide a conducive environment for detecting the subtle variations in postural sway that may be enhanced by the application of HAs. In contrast, the complexities introduced by varying surface conditions or closing the eyes could make it challenging to observe significant differences in postural sway. These observations underscore the importance of context in balance assessments, as the efficacy of HAs is likely contingent upon the stability of the environment and the sensory input available to the individual. Therefore, our findings indicate that while HAs are beneficial, their efficacy may be more pronounced in less challenging situations. This calls for further investigation into the specific conditions under which HAs can maximize their impact on postural control. Such conditions could include for example walking. In a previous study from our group, we found that stride-to-stride fluctuations (gait variability) increased during walking while counting backwards in older adults with hearing loss, but not in older adults without hearing loss. This suggests that walking could be a target of investigation for further studies (Kolasa et al., 2024).

The findings of the multiple regression analysis indicated that there was no significant association between hearing threshold and postural sway after adjusting for confounding factors. One possible explanation for the lack of a significant correlation in our study is that the range of HL in our sample (mild to moderate) may have been too narrow to detect a meaningful relationship between hearing threshold and postural sway. This factor should be considered in future studies to determine whether a wider range of HL would yield different results.

To sum up, the majority of existing research indicates a significant association between the use of HAs and enhanced balance; however, the effects varied across different tests and outcome measures (Malik et al., 2024; Lavie et al., 2024). The discrepancies in these findings may be attributed to methodological differences, particularly regarding the inclusion or exclusion of confounding factors and the assessment of hearing thresholds. Additionally, many studies did not provide sufficient detail regarding the use of HAs, including the duration and frequency (Malik et al., 2024; Lavie et al., 2024). Consequently, we concur with Lavie et al. (2024) that the current body of literature is inadequate to definitively ascertain the implications of HAs on postural sway. Our findings may have limited generalizability due to selection bias. The participants included in our study were highly educated, had few comorbidities, and did not exhibit reduced health and daily life function (WHODAS 12), signs of cognitive decline (TMT), or dizziness-related handicap (DHI-N) (Table 1). It is well known that individuals with higher education are healthier compared to those with lower educational attainment (Slade et al., 2020). These limitations should be considered when interpreting our findings and underscore the need for future studies.

A limitation is that we did not randomize the order of testing, which may have familiarized participants with the testing situation and thus improved their performance. Participants were tested with HAs first and without HAs second, potentially allowing them to familiarize themselves with the procedural elements of the balance evaluation, thereby enhancing their overall performance (Negahban and Nassadj, 2017). However, in the study by Negahban and Nassadj (2017), participants in the aided group underwent a series of postural tests initially with their HAs activated, followed by a second assessment with the devices deactivated, with interspersed five-minute resting period to mitigate any potential fatigue or adaptation effects, and they found similar results as we did. We did not specify the duration of HAs use or limit participation to experienced users, as our primary goal was to assess the overall impact of HAs on balance in older adults with HL. By including a broader sample, we aimed to identify the potential benefits of HAs, regardless of prior experience. However, we recognize that the duration of HAs use may affect balance outcomes, and future research could explore this factor further (Lubetzky et al., 2020).

In conclusion, our study indicates that older individuals with age-related hearing loss who use HAs maintain standing balance during quiet standing on a firm surface with eyes open, suggesting that HAs may enhance postural control and potentially reduce fall risk. We recommend that HAs use be considered in postural sway assessments. Our findings suggest that selection bias and order effects may influence the results of similar studies. We emphasize the need for future research to address these issues more rigorously by improving control over participant selection, ensuring a balanced testing order, and considering the long-term effects of HAs use on balance. These factors are important for gaining a clearer understanding of how HAs may influence balance and should be carefully examined in future studies.

## Data Availability

The original contributions presented in the study are included in the article/Supplementary material, further inquiries can be directed to the corresponding author.

## Footnotes

^1^ https://www.dagitty.net/
