# Supplementary material for: The effect of removing hearing aids on postural sway in older adults with age-related hearing loss: an experimental study
Source: Front Aging Neurosci. 2025 Mar 24;17:1534227. doi: 10.3389/fnagi.2025.1534227 (PMC11973377; doi:10.3389/fnagi.2025.1534227)

**Table 1s. Distribution of postural sway in persons with HL (N=50), without and with HAs.**

| Postural sway in millimeters (mm) | Hearing loss N=50 | |
| --- | --- | --- |
|  | Without hearing aids | With hearing aids |
| EOfirm, mean (SD), min-max | 283.16 (132.84)  83.8-625.6 | 243.01 (141.03)  80.7-963.4 |
| ECfirm, mean (SD), min-max | 527.64 (315.53)  155.6-1487.1 | 502.78 (400.60)  174.2-1831.5 |
| EOfoam, mean (SD), min-max | 395.76 (222.66)  171.8-1253.5 | 383.10 (202.72)  157.8-1539.2 |
| ECfoam, mean (SD), min-max | 1044.81 (420.76)  541.4-1930.4 | 1099.44 (493.46)  338.6-2477.8 |

*Abbreviations*: EOfirm = Postural sway on a firm surface with eyes open. ECfirm = Postural sway on a firm surface with eyes closed. EOfoam = Postural sway on a soft surface with eyes open. ECfoam = Postural sway on a soft surface with eyes closed.

**Graphs 1s. Scatterplots of the hearing threshold (PTA better ear) and log-transformed postural sway without HAs.**

a.
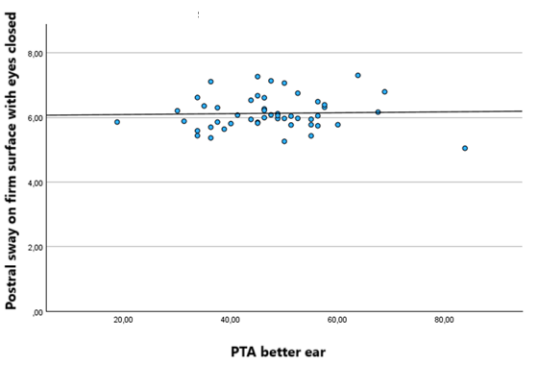


b.
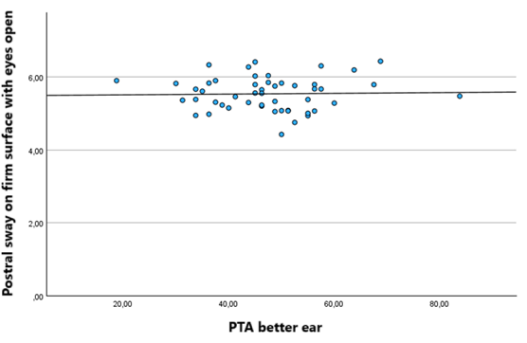


c.
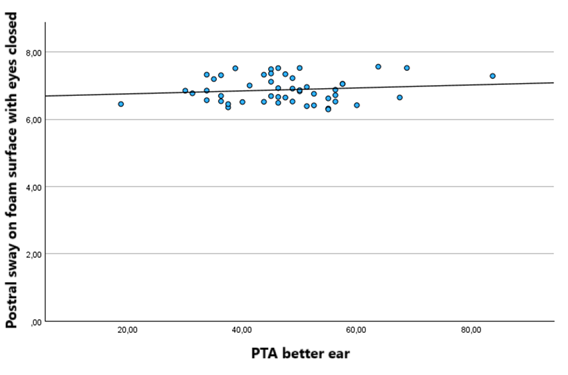


d.
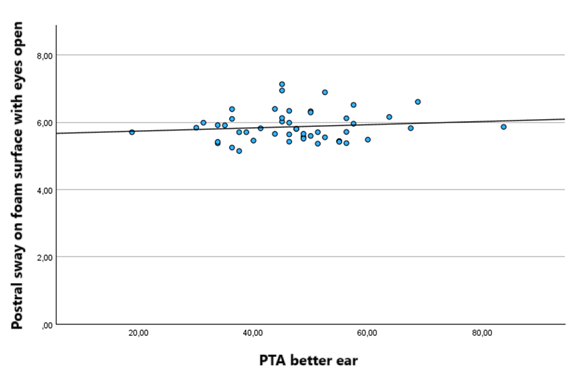


**Graphs 2s. Scatterplots of the hearing threshold (PTA better ear) and log-transformed postural sway with HAs.**

a.
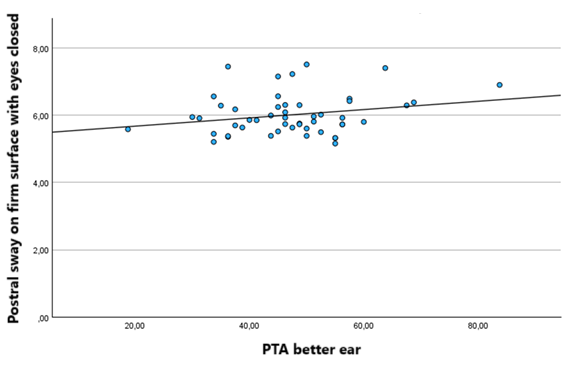


b.
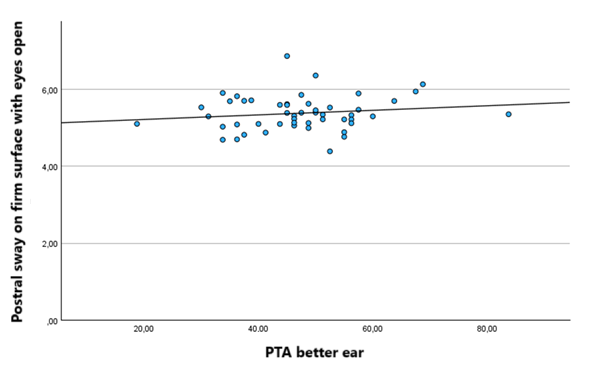


c.
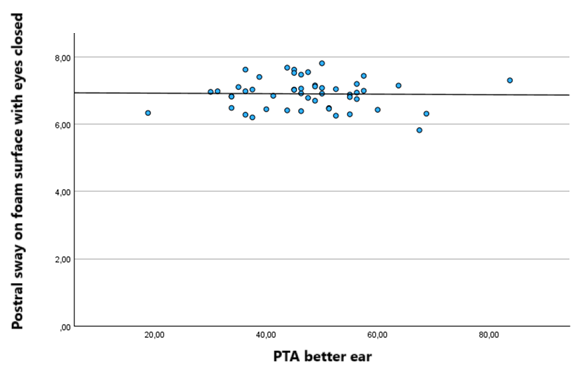


d.
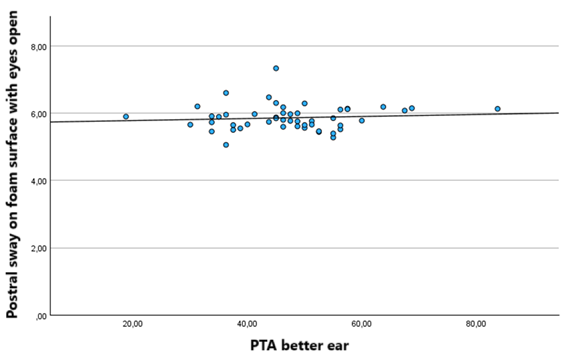

Supplement: Supplementary file 1 [file Data_Sheet_1_.docx]
